# Safety and effectiveness of benralizumab in Indian patients with severe eosinophilic asthma: results from the FAST study

**DOI:** 10.3389/fmed.2026.1706737

**Published:** 2026-04-20

**Authors:** Deepak Talwar, Venkata Nagarjuna Maturu, Priti L. Meshram, Saurabh Mittal, Janipalli Venkata Praveen, Randeep Guleria, Ashish Kumar, Ajay Agarwal, Ajay Kumar Verma, Murali Mohan, R. Narasimhan, Nitin Jain, Rahul Sharma, Rohit Kumar, Ujjwal Parakh, Gagandeep Momi

**Affiliations:** 1 Metro Hospitals and Heart Institute, Noida, India; 2 Yashodha Hospital, Hyderabad, Telangana, India; 3 Grant Government Medical College and Sir JJ Group of Hospital, Mumbai, Maharashtra, India; 4 All India Institute of Medical Science, New Delhi, India; 5 King George Hospital, Government Hospital for Chest and Communicable Diseases, Andhra Medical College, Visakhapatnam, India; 6 Medanta—The Medicity, Gurugram, Haryana, India; 7 Asthma Bhawan, Jaipur, Rajasthan, India; 8 Fortis Hospital, Noida, India; 9 King George's Medical University, Lucknow, India; 10 Narayana Hrudayalaya Institute of Medical Sciences, Bengaluru, Karnataka, India; 11 Apollo Hospital, Chennai, India; 12 Yatharth Super Speciality Hospitals, Noida, India; 13 VMMC and Safdarjung Hospital, New Delhi, India; 14 Sir Ganga Ram Hospital, New Delhi, India; 15 AstraZeneca Pharma India Ltd., Bengaluru, India

**Keywords:** benralizumab, effectiveness, eosinophilic severe asthma, India, postmarketing, safety

## Abstract

**Background and objective:**

The phase IV Fasenra Safety Trial in India (FAST) assessed the safety and effectiveness of benralizumab for a period of 24 weeks in adult Indian patients with severe eosinophilic asthma.

**Methods:**

This phase IV, single-arm, multicenter, prospective, interventional study (NCT05384938) included benralizumab-naïve adult patients (18–75 years) with a physician-confirmed diagnosis of severe asthma with an eosinophilic phenotype. Patients received 30 mg of benralizumab subcutaneously once every 4 weeks for the first three doses and then 30 mg once every 8 weeks thereafter as part of routine clinical care. The primary outcomes included adverse events (AEs), treatment-emergent adverse events (TEAEs), and serious TEAEs, along with the nature, incidence, and severity of AEs, including unexpected adverse drug reactions and AEs leading to treatment discontinuation or dose modifications. The secondary outcomes included time to first asthma exacerbation, annualized exacerbation rate, treatment outcome, and changes in absolute eosinophil count.

**Results:**

Of the 155 patients screened, 138 (89.0%) who received at least one dose of benralizumab were included in the safety and effectiveness analysis. At baseline, the median number of asthma exacerbation events per year was 2.0, and the median absolute eosinophil count was 375.0 cells/mm^3^ (range: 70.0–7352.8). Overall, 31.2% of the patients (43) experienced TEAEs; the most common TEAEs were pyrexia (16.7%), dyspnea (5.1%), productive cough (4.4%), cough (2.9%), and nasopharyngitis (2.2%). Serious TEAEs were reported in 5 (3.6%) patients and included dyspnea and productive cough (reported in 4 patients each), pyrexia (3), and constipation, H1N1 influenza, and back pain (1 each). No TEAEs leading to study drug discontinuation or death were reported. Nineteen (13.8%) patients experienced asthma exacerbation during the 24 weeks, with a median (range) time to first exacerbation event of 100.0 (41–184) days. A total of 61.5% of the patients (83/135) had well-controlled asthma, while 33.3% (45/135) had partly controlled asthma. A statistically significant decrease was observed in the mean asthma exacerbation events per year (from baseline to Week 24; 2.0 vs. 0; *p* < 0.0001) and peripheral blood eosinophil counts (from baseline to Weeks 4, 16, and 24; *p* < 0.001).

**Conclusion:**

The findings of this prospective, single-arm, multicenter, phase IV study in Indian patients with severe eosinophilic asthma demonstrated that benralizumab showed an acceptable and expected safety profile with consistent efficacy.

**Clinical trial registration:**

https://clinicaltrials.gov/study/NCT05384938, identifier NCT05384938.

## 1 Introduction

Severe asthma is a complex and serious form of asthma characterized by persistent symptoms that are difficult to control, even with high doses of standard medications. It significantly impacts daily life and often requires specialized treatment (1). Severe asthma can be primarily classified into eosinophilic and non-eosinophilic types based on airway or peripheral blood cellular profiles (2, 3). Severe asthma cases are classified into T2-high and T2-low, which represent distinct endotypes of asthma defined by the presence or absence of type 2 (T2) inflammation in the airways. T2-high asthma is characterized by increased levels of T2 inflammatory markers, such as eosinophils and type 2 cytokines, and typically responds well to biologic therapies targeting T2 cytokine pathways (4, 5). In contrast, T2-low asthma is frequently associated with neutrophilic or paucigranulocytic airway inflammation, lacks T2 cytokine-driven inflammation and eosinophilia, and is generally more difficult to treat with existing therapeutic options (6).

Severe asthma affects 5–8% of the Indian population, with a substantial proportion of cases being the eosinophilic type (49–64%) (7–9). Studies evaluating the prevalence and treatment practices of asthma in India have reported that 70% of the patients with severe asthma symptoms are not clinically diagnosed (10) and 46.3% of patients do not receive appropriate treatment (7), indicating underdiagnosis and undertreatment as major concerns in severe asthma management. Higher eosinophilic counts are frequently associated with asthma exacerbations, increased symptom burden, impaired lung function, a higher risk of hospitalization and mortality, and increased healthcare costs (11–18).

The current approach for the management of severe eosinophilic asthma is based on stepwise intensification of inhaled corticosteroids (ICS), along with add-on therapies, such as leukotriene receptor antagonists, long-acting beta2 agonists, long-acting muscarinic antagonists, anti-interleukin-4 (IL-4) receptor alpha subunit (IL-4Rα), anti-IL-5Rα, or anti-thymic stromal lymphopoietin therapies (19). Narasimhan (11) and Israel and Reddel (20) have reported that a substantial proportion of patients with severe or difficult-to-treat asthma remained poorly controlled despite maximal treatment with ICS and long-acting beta-agonists (LABAs). The Global Initiative for Asthma (GINA) guidelines recommend adding anti-IL-5Rα therapies for patients with severe eosinophilic asthma, depending on their availability and affordability (19).

Benralizumab is a humanized, afucosylated, monoclonal antibody that binds specifically to the human IL-5Rα subunit of eosinophils and basophils, thereby reducing airway inflammation by inhibiting eosinophilic recruitment and activation (21, 22). The pivotal SIROCCO (21), CALIMA (23), and ZONDA (24) trials have demonstrated the safety and efficacy of benralizumab in patients with severe, uncontrolled eosinophilic asthma. In addition, the MELTEMI trial has shown that the long-term use of benralizumab was safe and well-tolerated with no new safety signals in patients with severe, uncontrolled eosinophilic asthma, and demonstrated a similar reduction in exacerbation rate as observed in previous studies (25). The CALIMA (23) and SIROCCO (21) trials have shown a substantial reduction in exacerbations, along with improvements in lung function, asthma control, and quality of life, among benralizumab-treated patients with eosinophilic asthma, whereas the ZONDA (24) and PONENTE (26) trials have revealed significant reductions in daily oral corticosteroid doses. Additionally, the SHAMAL (27) trial has reported that benralizumab treatment resulted in substantial reductions in ICS therapy while maintaining asthma control in patients with eosinophilic asthma. Recently, Talwar et al. (28) shared their clinical experience with benralizumab in treating six Indian patients with severe eosinophilic asthma in a case series. The FAST study, a prospective interventional study, was designed to fulfill regulatory requirements following marketing approval for benralizumab in patients with severe eosinophilic asthma and to evaluate its safety over a 24-week period. This interventional study was based on the hypothesis that benralizumab treatment can effectively reduce eosinophilic airway inflammation and improve clinical outcomes in patients with severe eosinophilic asthma. The study also offers insights into the potential risks associated with eosinophil-lowering therapies when used in routine clinical practice in India, in accordance with locally approved label indications.

## 2 Methods

### 2.1 Study design

FAST (NCT05384938) was a phase 4, prospective, interventional, single-arm, multicenter study. The study protocol was approved by the independent ethics committees and institutional review boards of all participating centers before patient enrollment. The study was conducted in compliance with the study protocol, the Declaration of Helsinki, the International Council for Harmonisation, the Good Clinical Practices Guideline, the Council for International Organizations of Medical Sciences International Ethical Guidelines, and the relevant regulations governing interventional studies. Given the study design, the reporting of this manuscript has been prepared in accordance with the Strengthening the Reporting of Observational Studies in Epidemiology (STROBE) checklist (29).

### 2.2 Study population

Benralizumab-naïve adult patients (18–75 years of age) with a physician-confirmed diagnosis of severe asthma with an eosinophilic phenotype (eosinophil count of ≥300 cells/μL in the 12 months preceding study screening); those with a decreased lung function requiring high doses of ICS (>500 μg fluticasone propionate dry powder formulation, >800 μg budesonide dry powder formulation, or equivalent total daily dose) and a long-acting beta-agonist (LABA) as maintenance treatment for at least 3 months before enrollment, with at least two exacerbations in the preceding 12 months; and those who were prescribed benralizumab (30 mg, subcutaneous injection) as an add-on therapy as part of routine clinical care and were scheduled to initiate benralizumab treatment within 2 weeks were included in the study. Patients were excluded if they had clinically important pulmonary or systemic disease other than asthma, which could confound the outcome assessment; if they were enrolled in an interventional clinical study concurrent with this study, including those involving biologic treatments; or if they had received any biologic therapy within 30 days before the date of informed consent. Supplementary Table 1 provides the detailed list of inclusion and exclusion criteria for this study.

### 2.3 Study procedures and data collection

Patients were screened for eligibility up to 2 weeks before study initiation. Written informed consent was obtained from all patients before screening for eligibility. Enrolled patients were administered 30 mg of benralizumab subcutaneously (upper arm, thigh, or abdomen) once every 4 weeks for the first three doses and then 30 mg subcutaneously every 8 weeks. Benralizumab was made available for the study by AstraZeneca Pharma India Ltd. The study duration was 24 weeks, with 4 scheduled visits at Day 1, Week 4, Week 8, and Week 16 for benralizumab administration and safety assessments, and a post-treatment follow-up visit at Week 24 (Supplementary Figure 1).

Data were collected from the electronic case report forms of the patients at the sites and included demographics, baseline pre- and post-bronchodilator forced expiratory volume in 1 s (FEV1), and relevant medical history. Clinical safety laboratory assessments (chemistry, hematology, and urinalysis) were conducted at baseline and Week 24, while electrocardiograms (ECGs) were performed at baseline, Week 4, and Week 24. Peripheral blood absolute eosinophil count was measured at baseline, Week 4, Week 16, and Week 24. Asthma exacerbations were assessed at baseline, Week 4, Week 8, Week 16, and Week 24. Additionally, vital signs, physical examinations, adverse events (AEs), and concomitant asthma medications were recorded at baseline and at each subsequent visit (Supplementary Figure 1).

### 2.4 Study measures and outcomes

The primary outcomes included AEs, treatment-emergent adverse events (TEAEs), serious TEAEs, and the nature, incidence, and severity of AEs, including unexpected adverse drug reactions and TEAEs leading to study treatment dose discontinuation/modifications. AEs were coded by system organ class and preferred term according to the Medical Dictionary for Regulatory Activities version 24.1. The secondary outcomes included time to first asthma exacerbation, annualized exacerbation rate, treatment outcome (level of asthma control assessed by the investigator as well-controlled, partly controlled, or uncontrolled asthma), and change in absolute eosinophil count. Detailed safety and effectiveness assessments are provided in Supplementary Table 2.

### 2.5 Statistical analyses

Based on the incidence of key AEs with benralizumab, which ranges from 1 to 10% (Supplementary Table 3) (21, 22), approximately 139 patients were required to provide a 95% confidence interval (CI) around the estimated AE incidence rate with an absolute precision level of 0.05. Considering a dropout rate of 5%, a sample size of 147 patients was planned for the study. Safety analyses were performed using the safety analysis set—all patients who received at least one dose of benralizumab. Effectiveness analyses were performed using an evaluable analysis set—all patients who received at least one dose of benralizumab and had at least one post-baseline assessment. Statistical analyses were performed using SAS version 9.4 or higher (SAS Institute Inc., NC, USA). Categorical variables were summarized as frequencies, percentages, and their corresponding 95% confidence intervals (CIs; using the Clopper–Pearson exact method). Continuous variables were summarized as mean, standard deviation, median, and range. The time-to-event endpoint was summarized using the Kaplan–Meier (KM) method with the corresponding 95% CIs. A *p*-value of <0.05 was considered statistically significant.

## 3 Results

**Figure 1:**
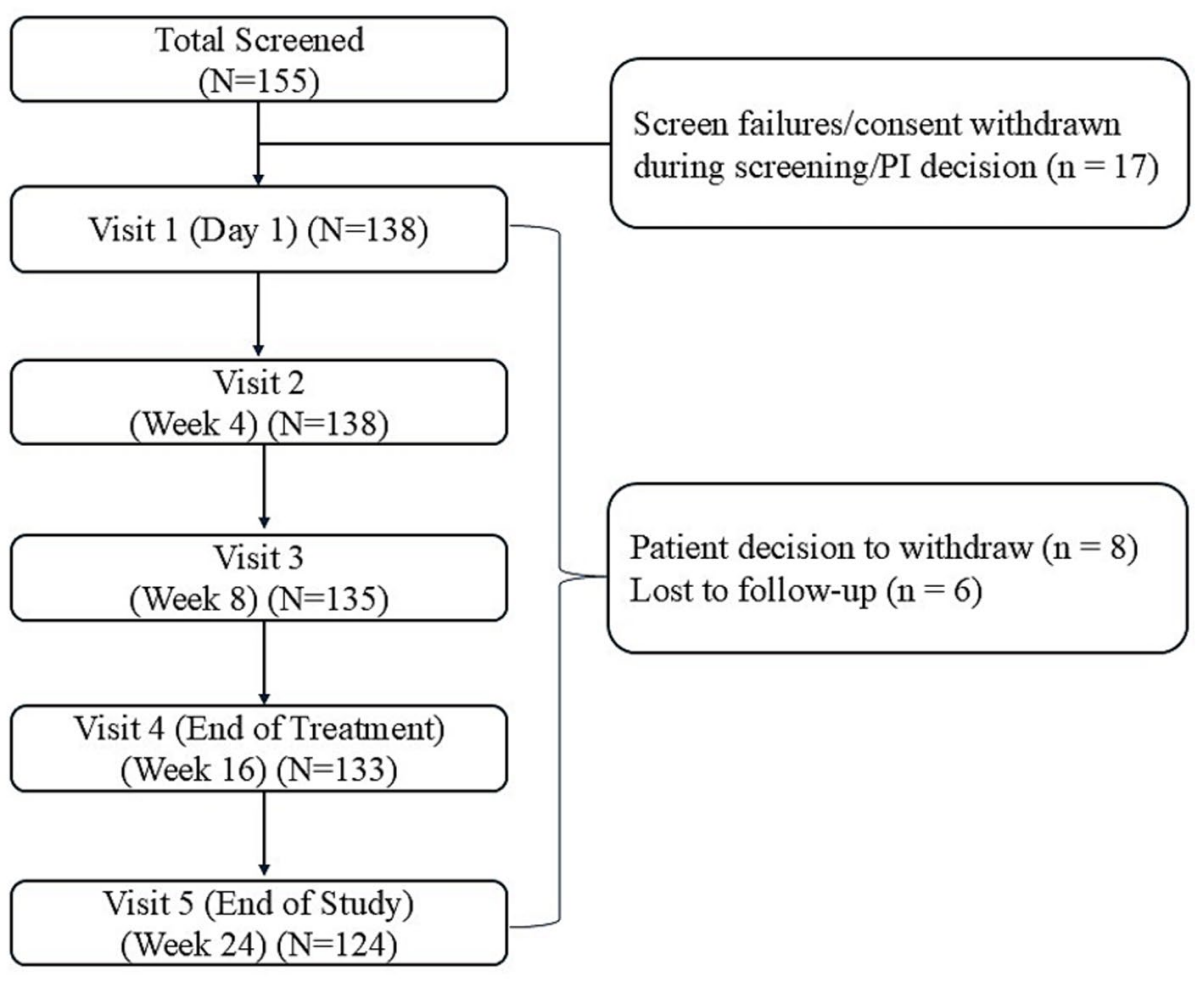
Patient disposition and reasons for discontinuation of benralizumab in the study. PI, principal investigator; *N*/*n*, number of patients. Benralizumab was administered subcutaneously at Visit 1 (Day 1), Visit 2 (Week 4), Visit 3 (Week 8), and Visit 4 (Week 16). A post-treatment follow-up visit was performed at Week 24.

The FAST study was conducted between November 2021 and July 2023 at 13 centers across India. A total of 155 patients were screened, of whom 11 did not pass screening, 5 withdrew consent before drug administration, and 1 patient was excluded due to an abnormal ECG (Figure 1).

A total of 138 (89.0%) patients who received at least 1 dose of benralizumab were included in the safety and effectiveness analysis. Of these patients, 133 (96.4%) received all 4 doses of benralizumab, 124 (89.9%) completed the study (Visit 5, end-of-study visit), and 14 (10.1%) discontinued the study (Figure 1).

### 3.1 Baseline characteristics

The median (range) age of the patients was 47.0 years (20.0–75.0); 71 (51.5%) were female (Table 1). The median (range) asthma duration was 6.8 years (1.1–60), and the mean body mass index was 25.7 ± 5.2 kg/m^2^. All 138 (100%) patients received prior asthma medications and oral corticosteroids/ICS. The median (range) pre-bronchodilator FEV1 was 1.2 L (0.4–2.9), the median (range) pre-bronchodilator predicted normal FEV1 was 47.0% (18.0–79.0), and the median (range) pre-bronchodilator forced vital capacity was 2.0 L (0.7–3.9). The median number of asthma exacerbation events per year was 2.0 (range: 2, 2). The median absolute eosinophil count was 375.0 cells/mm^3^ (range: 70.0–7352.8).

**Table 1:** Demographics and baseline characteristics.

Parameters	*N* = 138
Age (years), median (range)	47.0 (20.0–75.0)
Female, *n* (%)	71 (51.5)
Body mass index (kg/m^2^), mean (SD)	25.7 (5.2)
Asthma duration (years), median (range)	6.8 (1.1–60)
Blood eosinophil count (cells/mm^3^) at baseline, median (range)	375.0 (70.0–7352.8)
Number of exacerbations in the past	276
Pre-bronchodilator assessment at baseline	
FEV1 (L), median (range)	1.2 (0.4–2.9)
Predicted normal FEV1 (%), median (range)	47.0 (18.0–79.0)
Forced vital capacity (L), median (range)	2.0 (0.7–3.9)
Post-bronchodilator reversibility at baseline, *n* (%)	46 (33.3)
FEV1 (L), median (range)	1.4 (0.6–3.6)
Reversibility FEV1 (%), median (range)	29.0 (12.0–97.0)
FEV1 reversibility (mL), median (range)	300.0 (150.0–920.0)
Patient with any medical history, *n* (%)	33 (23.9)
Surgical and medical procedures	8 (5.8)
Gastroesophageal reflux disease	7 (5.1)
Type 2 diabetes mellitus	7 (5.1)
Rhinitis allergic	6 (4.4)
Migraine	2 (1.5)
Hypothyroidism	1 (0.7)
Insomnia	1 (0.7)
Hypertension	1 (0.7)
COVID-19 vaccination status, *n* (%)	
No	17 (12.3)
Yes	121 (87.7)
1st dose of COVID-19 vaccine	121 (100.0)
2nd dose of COVID-19 vaccine	107 (88.4)
3rd dose of COVID-19 vaccine	22 (18.2)

COVID-19, coronavirus 2019; FEV1, forced expiratory volume in 1 s; *n*, number of patients; SD, standard deviation.

### 3.2 Safety assessments

**Figure 2:**
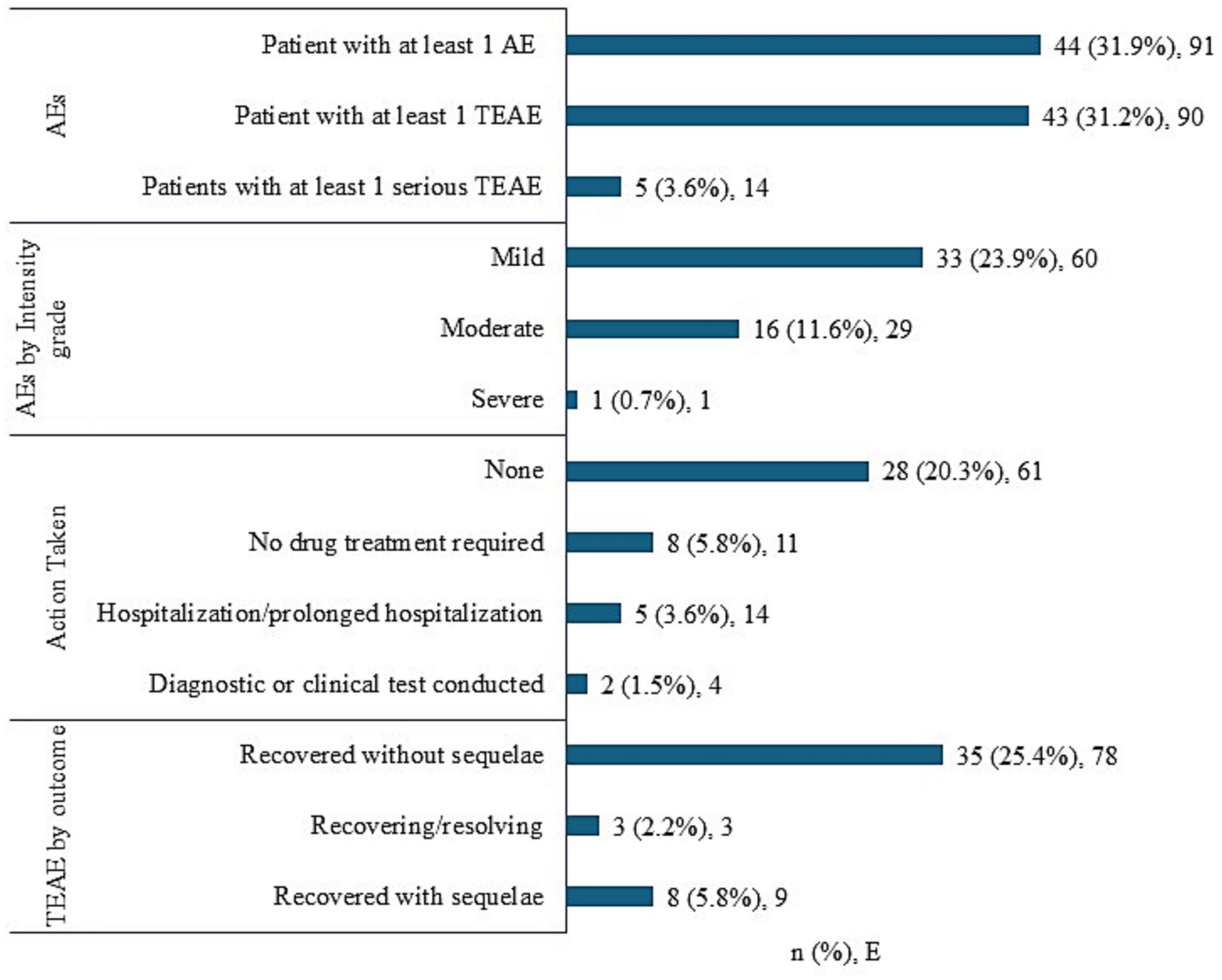
Summary of adverse events. AEs, adverse events; *E*, number of TEAE; *n*, number of patients; TEAEs, treatment-emergent adverse events. AEs were coded using the Medical Dictionary for Regulatory Activities (version 24.1). TEAEs were those AEs that occurred after the first dose of study medication. If a patient had multiple occurrences of an AE, the patient was presented only once. Events were counted each time at their occurrence.

A total of 43 (31.2%) patients experienced 90 TEAEs. Mild TEAEs were reported in 33 (23.9%) patients, and 16 (11.6%) patients had moderate TEAEs, while only 1 (0.7%) patient experienced a severe TEAE. Five (3.6%) patients experienced serious TEAEs requiring hospitalization; all of the five patients recovered without any sequelae. Overall, 35 patients recovered from the events without any sequelae (25.4%), 8 (5.8%) recovered with sequelae, and 3 (2.2%) were still recovering at the end of the study. No TEAEs leading to study drug discontinuation or death were reported (Figure 2).

**Figure 3:**
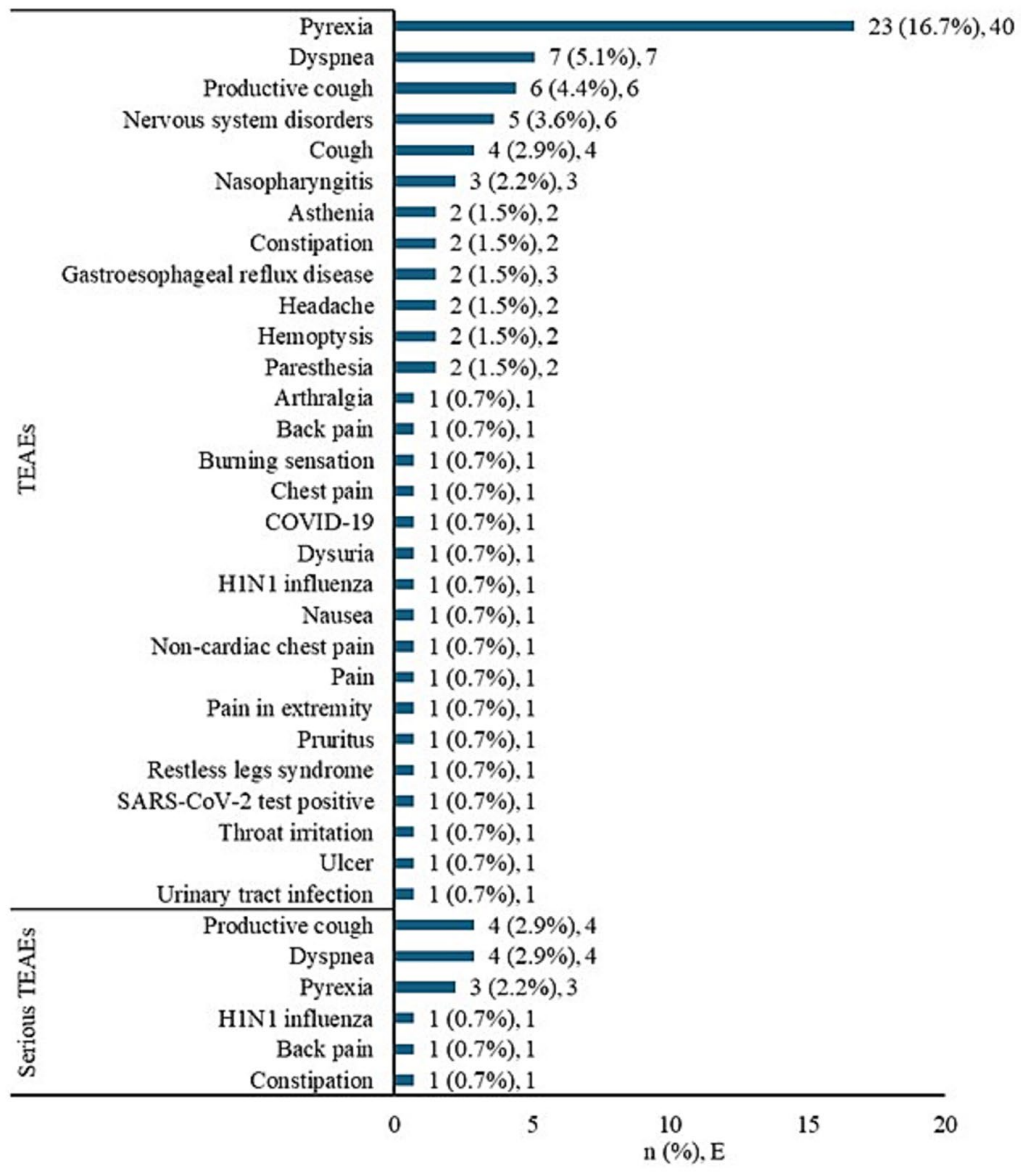
TEAEs and serious TEAEs by preferred term. *E*, number of TEAE; *n*, number of patients; TEAEs, treatment-emergent adverse events. Note: Adverse events were coded using the Medical Dictionary for Regulatory Activities (version 24.1). TEAEs were those adverse events that occurred after the first dose of study medication. Drug-related TEAEs were reported for two patients (1.5%, *E* = 2); the preferred term was pyrexia. If a patient had multiple occurrences of an AE, the patient was presented only once. Events were counted each time at their occurrence.

The TEAEs that occurred in >2% of patients were pyrexia (16.7%), dyspnea (5.1%), productive cough (4.4%), cough (2.9%), and nasopharyngitis (2.2%) (Figure 3). Pyrexia was reported in 1.5% of patients and was deemed causally related to benralizumab treatment. Serious TEAEs included dyspnea (2.9%), productive cough (2.9%), pyrexia (2.2%), constipation (0.7%), H1N1 influenza (0.7%), and back pain (0.7%) (Figure 3).

No clinically significant changes in hematology, clinical chemistry (renal and hepatic), urinalysis, physical examinations, vital signs, or ECGs were reported during the entire study period (Supplementary Table 4).

### 3.3 Effectiveness assessments

**Figure 4:**
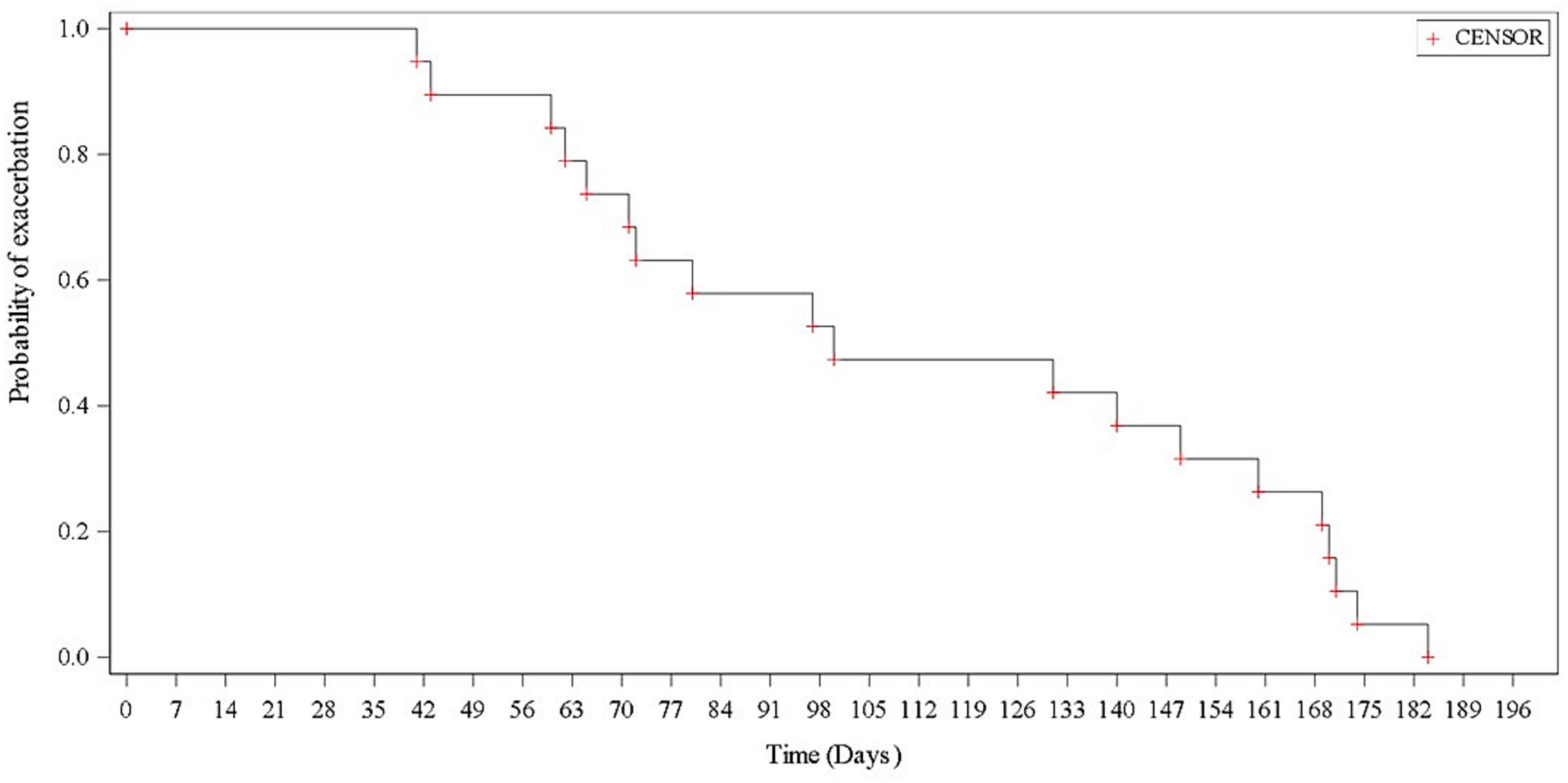
The Kaplan–Meier plot showing time to first asthma exacerbation (in days).

**Figure 5:**
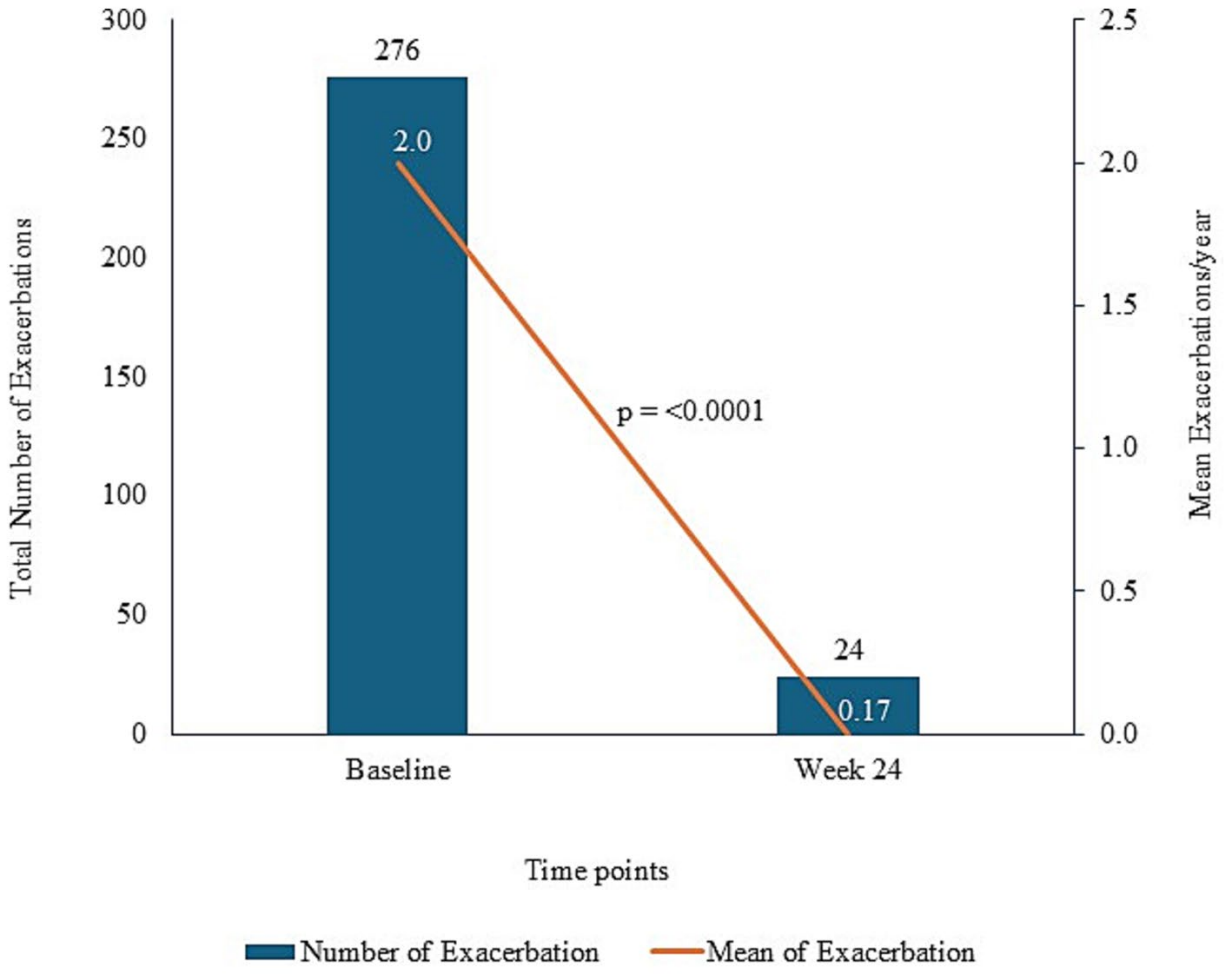
Exacerbation frequency and mean exacerbation events at baseline and week 24 after benralizumab treatment.

A total of 19 (13.8%) of 138 patients experienced asthma exacerbation during the study period. The median (range) time to the first exacerbation event after the first dose of benralizumab was 100.0 (41–184) days. Asthma exacerbation of clinical significance as per the investigator’s assessment was reported in 9 patients, of which 3 required an emergency room visit and 3 required hospitalizations. The KM estimate for the median time to first asthma exacerbation after the first dose of benralizumab was 100.0 days (95% CI: 65–160) (Figure 4). There was a statistically significant decrease in mean asthma exacerbation events per year from 2.0 at baseline to 0.17 at Week 24 (*p* = 0.0001; exacerbation events: 276 at baseline and 24 at Week 24) (Figure 5). The annualized exacerbation rate at Week 24 was 0.36.

The investigator’s assessment for asthma control was performed in 135 patients, with the majority of patients (83, 61.5%) being classified as having “well controlled” asthma; 45 (33.3%) and 7 (5.2%) were classified as “partly controlled” and “uncontrolled,” respectively. Figure 5 presents changes in the number of exacerbations and the mean exacerbation events per year at baseline and at Week 24 after benralizumab treatment. The median absolute eosinophil count decreased to 50.0 cells (range: 0–1,252) cells/mm^3^ at Week 24 from a baseline value of 375.0 cells/mm^3^ (range: 70.0–7352.8). We observed a statistically significant reduction in peripheral blood eosinophil counts from baseline to Week 4 (median change, −292; range, −7257.0–189.0), Week 16 (median change, −308; range, −7306.0 to 626.0), and Week 24 (median change, −306; range, −7353.0–599.0) (*p* = 0.0001) (Table 2).

**Table 2:** Change in peripheral blood eosinophil count.

Time	Blood eosinophil count, cells/mm^3^	Change from baseline, median (range)	*p*-Value	% Change from baseline, median (range)
Baseline, *N* = 138	375.0 (70.0, 7352.8)			
Week 4, *N* = 138	70.0 (0, 5788.0)	−292 (−7257.0, 189.0)	<0.0001	−86.1 (−100, 217.2)
Week 16, *N* = 133	56.0 (0, 1242.0)	−308 (−7306.0, 626.0)	<0.0001	−91.8 (−100, 176.8)
Week 24, *N* = 124	50.0 (0,1252.0)	−306 (−7353.0, 599.0)	<0.0001	−92.4 (−100.0, 234.9)

*N*/*n*, number of patients.

## 4 Discussion

The FAST is the first prospective interventional study to generate data on the safety, tolerability, and effectiveness of benralizumab in patients with severe eosinophilic asthma who were prescribed the treatment as part of routine clinical care following its regulatory approval in India. The results indicate that benralizumab is safe and well-tolerated in Indian patients, with no new safety signals.

In our study, the median asthma duration was 6.8 years. The median pre-bronchodilator predicted normal FEV1 was 47.0%. The median pre-bronchodilator and post-bronchodilator FEV1 were 1.2 L and 1.4 L, respectively. Similar findings, with a pre-bronchodilator FEV1 of 1.9 L and pre-bronchodilator predicted FEV1 of 67.3%, were reported in a real-world observational study by Yamaguchi et al. (30) from Japan.

Overall, 31.2% of the patients experienced at least 1 TEAE (90 TEAEs); serious TEAEs were reported in 3.6% of the patients, and drug-related TEAEs were reported in 1.5% of patients. The majority of these TEAEs (78 of 90 events) resolved without sequelae. Conversely, a real-world Japanese study (*N* = 632) by Yamaguchi et al. reported adverse drug reactions in 12.7% of the patients, and 13.0% of the patients experienced serious adverse events (30). A recent case series of 6 patients from India by Talwar et al. (28) reported that benralizumab treatment was tolerated without any clinically significant adverse events. Pivotal trials such as SIROCCO, CALIMA, BORA, and the BORA extension also reported a higher rate of TEAEs (70.7 to 86.2% vs. 31.2%), along with TEAEs leading to death (0.3 to 0.6% vs. 0%) and treatment discontinuations (0.9 to 2.2% vs. 0%), compared with our study (21, 31). Real-world studies assessing the efficacy and safety of benralizumab in Japanese patients with severe eosinophilic asthma reported a higher overall discontinuation rate of 32.0–41% (30, 32). However, no TEAEs leading to death or discontinuation of the study drug were reported in our study. The most common TEAEs reported were pyrexia, dyspnea, and nasopharyngitis, which were consistent with the published literature, although with differences in the frequencies (21, 23, 30). Other common TEAEs reported with benralizumab, such as asthma, bronchitis, sinusitis, viral upper respiratory tract infection, and pneumonia, were not reported in our study (21, 25, 30, 33). Our study also did not report the adverse events of interest, such as hypersensitivity and injection site reactions, which were previously reported by Lai et al. (33).

Currently, based on the data from pivotal trials, such as SIROCCO (21), CALIMA (23), and ZONDA (24), add-on benralizumab is recommended to be included in the standard of care in patients with severe eosinophilic asthma by international (19) guidelines and by recommendations from the Indian Medical Association, based on its availability and accessibility (34). Several real-world studies from various countries have reported the safety and effectiveness of add-on benralizumab for the management of severe eosinophilic asthma (30, 32, 35, 36). In the past 4 years after the approval of benralizumab in India, only one clinical experience, reported as a case series by Talwar et al. (28), has been published. Our study reported a decrease in exacerbation frequency (from a total of 276 to 24 exacerbation events) and mean exacerbation events (2.0 to 1.17; *p* < 0.05) with benralizumab treatment. The median time to first asthma exacerbation was 100 days. Nearly 95% of patients achieved better control of asthma according to the investigator’s assessment. Peripheral blood eosinophil counts were significantly suppressed after 4 weeks of benralizumab treatment, and the suppression was maintained throughout the observation period (from 375 cells/mm^3^ to 50 cells/mm^3^; *p* = 0.0001) in our study, which is consistent with the studies that assessed the effect of benralizumab in patients with severe eosinophilic asthma (25, 37). Pelaia et al. (37) investigated the therapeutic effects of benralizumab in Italian patients with severe, uncontrolled, corticosteroid-refractory eosinophilic asthma and reported that add-on benralizumab treatment resulted in significant depletion of peripheral blood eosinophils, along with a decrease in the number of asthma exacerbations and improved asthma control after 24 weeks of treatment, similar to our study. A case series of six patients from India by Talwar et al. (28) demonstrated a marked improvement in lung function and quality of life, a reduction in eosinophil count, decreased hospitalizations, and an oral corticosteroid tapering effect (tapered/discontinuation) with an acceptable safety profile. MIRACLE, a randomized trial from Asia, reported that benralizumab-treated patients had a longer time to first asthma exacerbation than those who received placebo, as indicated by a reduced risk of exacerbation [hazard ratio (HR), 0.32; (95% CI, 0.23–0.43); nominal *p* < 0.0001] (33). Lai et al. (33) also showed that benralizumab reduced the annual asthma exacerbation rate associated with emergency or urgent hospitalizations by 54% compared with placebo (rate ratio, 0.46; 95% CI, 0.24–0.90; nominal *p* = 0.0222).

To the best of our knowledge, this is the first Indian study to demonstrate that benralizumab is well-tolerated, presents no new safety signals, and effectively reduces mean exacerbation events, along with eosinophil counts, in patients with severe eosinophilic asthma.

Similar to other phase IV studies, this study was limited by a small sample size, a short follow-up period, and the absence of an assessment of improvements in lung function and quality of life. Additionally, the absence of a control group prevented a direct comparison of the effectiveness of benralizumab with standard care. Furthermore, the use of rescue oral corticosteroids in exacerbation cases was not evaluated.

## 5 Conclusion

The FAST study results confirm that the safety and tolerability of benralizumab align with its established safety and efficacy profile from Phase III trials and real-world data. Benralizumab was well-tolerated with no new safety concerns. While our study results will help guide treatment decisions for benralizumab in patients with severe eosinophilic asthma, long-term safety and effectiveness data, combined with broader clinical use, will further reinforce its role in managing this patient population.

## Data Availability

Data underlying the findings described in this manuscript may be obtained in accordance with AstraZeneca’s data-sharing policy described at https://astrazenecagrouptrials.pharmacm.com/ST/Submission/Disclosure.
