# Supplementary material for: Safety and effectiveness of benralizumab in Indian patients with severe eosinophilic asthma: results from the FAST study
Source: Front Med (Lausanne). 2026 Apr 20;13:1706737. doi: 10.3389/fmed.2026.1706737 (PMC13136968; doi:10.3389/fmed.2026.1706737)
Supplement: Supplementary file 1 [file Data_sheet_1.pdf]

Supplementary Material

Safety and Effectiveness of Benralizumab in Indian Patients with Severe Eosinophilic Asthma: Results from FAST Study

Deepak Talwar[1*], Venkata Nagarjuna Maturu[2], Priti L Meshram[3], Saurabh Mittal[4], Janipalli Venkata Praveen[5], Randeep Guleria[6], Ashish Kumar[7], Ajay Agarwal[8], Ajay Kumar Verma[9], Murali Mohan[10], Narasimhan R[11], Nitin Jain[12], Rahul Sharma[13], Rohit Kumar[14], Ujjwal Parakh[15], Gagandeep Momi[16]

***Correspondence:** Dr Deepak Talwar; Email address: dtlung@gmail.com

1. Supplementary Figure

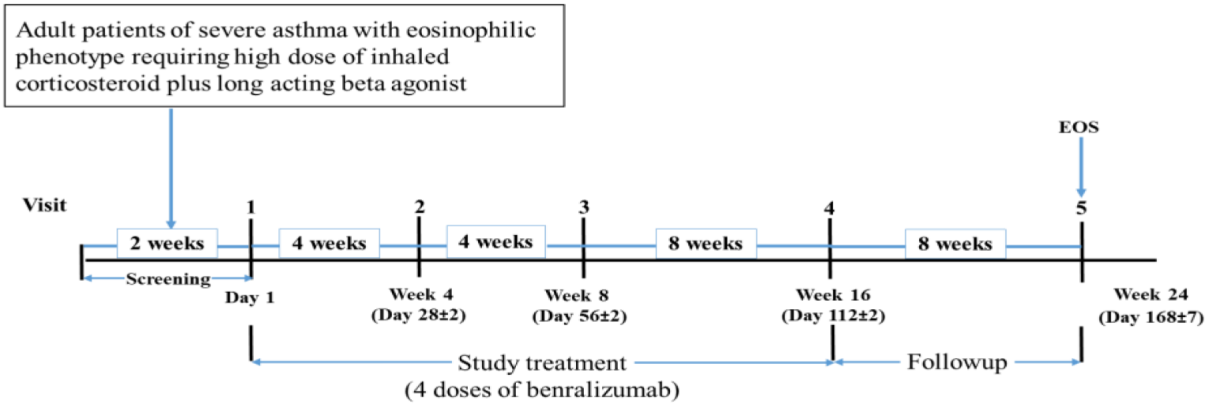

EOS, end of study.

Supplementary Figure 1. Flow chart of study design

2. Supplementary Tables

Supplementary Table 1. Detailed eligibility criteria of FAST study population

	Eligibility Criteria
**Inclusion Criteria**	• Male or female patients 18 to 75 years of age inclusive, at the time of signing the informed consent.<br>• Patients with physician's confirmed diagnosis of severe asthma with an eosinophilic phenotype, ie, a diagnosis of severe asthma in preceding at least 12 months, with an eosinophil count of ≥300 cells/μL at screening, requiring treatment with high-dose ICS (>500 μg fluticasone propionate dry powder formulation, or >800 μg budesonide dry powder formulation, or equivalent total daily dose) and a LABA as maintenance treatment for at least 3 months prior to enrollment.<br>• A decreased lung function with prebronchodilator FEV1 of <80% predicted, demonstrated by spirometry at screening.<br>• At least 2 documented asthma exacerbations in the preceeding12 months, except in 30 days before the date of informed consent, that required the use of a systemic corticosteroid or temporary increase from the patient's usual maintenance dose of oral corticosteroid.<br>• Documented postbronchodilator reversibility in FEV1 of ≥12% and ≥200 mL in FEV1 within 12 months before first dose. If historical documentation is not available, reversibility must be demonstrated and documented at screening or Day 1 before the first dose.<br>• Benralizumab naïve patients who have not previously received benralizumab prior to the start of this study.<br>• Patients who are willing and capable of giving signed informed consent, which includes compliance with the requirements and restrictions listed in the informed consent form and in this protocol.
**Exclusion Criteria**	• Clinically important pulmonary disease other than asthma (eg, active lung infection, chronic obstructive pulmonary disease, bronchiectasis, pulmonary fibrosis, cystic fibrosis etc.) or ever been diagnosed with pulmonary or systemic disease, other than asthma, that are associated with elevated peripheral eosinophil counts (eg, allergic bronchopulmonary aspergillosis/mycosis, Churg-Strauss syndrome, hypereosinophilic syndrome), which can confound the outcome assessment.<br>• Patients currently enrolled in an interventional clinical study in parallel including those with any biologic treatment.<br>• Patients who have received any biologic within 30 days prior to the date of informed consent.<br>• Known history of allergy or reaction to the benralizumab formulation or excipients (L-histidine, L-histidine hydrochloride monohydrate, α-trehalose dihydrate, polysorbate 20, water for injection).<br>• History of anaphylaxis to any biologic therapy.<br>• A helminth parasitic infection diagnosed within 24 weeks before the date informed consent is obtained that has not been treated with, or has failed to respond to, standard of care therapy.<br>• Acute asthma exacerbation 30 days before the date of informed consent.<br>• Acute asthma exacerbation between screening and first dose of study dose administration.<br>• Acute upper or lower respiratory infections requiring antibiotics or antiviral medication within 30 days before the date of informed consent.<br>• Patients with a malignancy within 5 years prior to enrollment, with the exception of adequately treated in-situ carcinoma of the cervix, uteri, basal, or squamous cell carcinoma, or non-melanomatous skin cancer with active or recent malignancy.

	• Any clinically significant abnormal findings in physical examination, vital signs, hematology, clinical chemistry, or urinalysis, which, in the opinion of the investigator, may put the participant at risk because of his/her participation in the study.<br>• History of current alcohol, drug, or chemical abuse or past abuse that would impair or risk the participant's full participation in the study, in the opinion of the investigator.<br>• Female patients who are pregnant or lactating or planning a family during the study period.

FEV1, forced expiratory volume in 1 second; ICS, inhaled corticosteroids; LABA, long-acting beta-agonist

Supplementary Table 2: Safety and effectiveness assessments

Safety Assessments	
Treatment-emergent adverse event	A treatment-emergent adverse event was defined as any event that does not present prior to the initiation of the drug treatment or any event already present that worsens in either intensity or frequency following exposure to the drug treatment.
Serious adverse events	A serious adverse event was an adverse event occurring during any study phase that fulfilled one or more of the following criteria:<br>• Results in death<br>• Immediately life-threatening<br>• Required in-participant hospitalization or prolongation of existing hospitalization<br>• Resulted in persistent or significant disability or incapacity<br>• Led to congenital abnormality or birth defect<br>• Important medical event that may jeopardize the participant or may require medical treatment to prevent one of the outcomes listed above
Severity	• Mild: Usually transient and might require only minimal treatment or therapeutic intervention. The event did not generally interfere with usual activities of daily living.<br>• Moderate: Usually alleviated with additional specific therapeutic intervention. The event interfered with usual activities of daily living, causing discomfort but poses no significant or permanent risk of harm to the patient.<br>• Severe: Interrupted usual activities of daily living or significantly affected clinical status or might require intensive therapeutic intervention.
Causality/Relatedness	The investigator assessed a causal relationship between benralizumab and each adverse event and serious adverse event based on the time course (exposure to suspect drug), consistency with known drug profile, de-challenge experience, no alternative cause, re-challenge experience, laboratory tests, and other factors (recognized feature of overdose of the drug and known mechanism of action).<br>The causal relationship in cases where the disease under study deteriorated due to lack of effect was classified as no reasonable possibility.
Vital signs	Vital signs (pulse, blood pressure, respiration rate, and body temperature) and electrocardiogram were taken before benralizumab administration, and, if possible, blood drawing and usual asthma controller medication.
Physical examinations	A targeted physical examination was performed, including assessments of the

	following: general appearance, respiratory, cardiovascular, abdomen, skin, head and neck (including ears, eyes, nose, and throat), lymph nodes, thyroid, musculoskeletal (including spine and extremities), and neurological systems.
Effectiveness Assessments	
Asthma exacerbations	Asthma exacerbation was defined as a worsening of asthma that leads to any of the following:<br>• Use of systemic corticosteroids (or a temporary increase in a stable oral corticosteroids background dose) for at least 3 days; a single depot-injectable dose of corticosteroids was considered equivalent to a 3-day course of systemic corticosteroids.<br>• An emergency room/urgent care visit (defined as evaluation and treatment for <24 hours in an emergency department or urgent care center) due to asthma that required systemic corticosteroids<br>• An inpatient hospitalization (defined as admission to an inpatient facility and/or evaluation and treatment in a healthcare facility for ≥24 hours) due to asthma.
Time to first asthma exacerbation	It was calculated from baseline (Day 1) to time of first exacerbation. The start of an exacerbation was defined as the start date of systemic corticosteroids, emergency room/urgent care visits requiring systemic corticosteroids, or hospital admissions due to asthma, whichever occurs earlier. The end date was defined as the last day of systemic corticosteroids or emergency room/urgent care/hospital discharge, whichever occurs later.
Number of exacerbations experienced by a patient	The start of an exacerbation was defined as the start date of systemic corticosteroids or start date of a temporary increase in a stable oral corticosteroids background dose, or start date of hospital admission, whichever occurred earlier. The end date was defined as the last day of systemic corticosteroids or the last day of a temporary increase in a stable oral corticosteroids background dose, or the date of discharge from a hospital, whichever occurs later.
Prebronchodilator FEV1	Prebronchodilator forced expiratory volume in 1 second was recorded by spirometry and performed at screening to confirm the diagnosis of severe asthma.
Annual exacerbation rate	The annual exacerbation rate for each patient was calculated by dividing the total number of exacerbations by the number of days participated in the study and multiplying by 365.

FEV1, forced expiratory volume in 1 second

Supplementary Table 3: Assumptions for sample size

	**Incidence Rate**		
**Key Adverse Events**	**SmPC/ India Label**	**USPI**(22)	**SIROCCO**(21)
Headache	Common (1%-10%)	8%	7%-9%
Pharyngitis		5%	4%-6%
Pyrexia		3%	3%-4%
Injection site reactions		2.2%	2%-4%
Hypersensitivity		3%	3%

SmPC, summary of product characteristics; USPI, United States prescribing information

Supplementary Table 4: Changes in hematology and clinical chemistry parameters from baseline to end of study

Parameter	Safety Analysis Set (N=138), mean (SD)				
		Week 24			
	Observed value at baseline	Observed value	Change from baseline	p-value	% Change from baseline
Hematocrit (%)	41.3 (5.8)	40.5 (4.8)	-0.6 (3.9)	0.1111	-0.3 (13.1)
Hemoglobin (g/dL)	13.4 (2.0)	13.1 (1.7)	-0.3 (1.2)	0.0063	-1.3 (12.6)
Absolute neutrophils count (mm^3)	5545.8 (2100.3)	5059.5 (2337.6)	-446.7 (2197.0)	0.0253	-2.7 (37.6)
Absolute basophils count (mm^3)	22.0 (35.1)	13.3 (22.1)	-9.3 (30.7)	0.0010	-36.0 (68.5)
Absolute eosinophils count (mm^3)	639.5 (908.7)	103.9 (195.0)	-540.6 (893.6)	<0.0001	-71.5 (55.8)
Absolute monocytes count (mm^3)	581.1 (243.3)	542.7 (281.1)	-44.6 (244.0)	0.0438	-1.4 (47.3)
Absolute lymphocytes count (mm^3)	2476.1 (928.5)	2227.3 (725.4)	-267.3 (786.5)	0.0002	-4.1 (34.0)
Platelet count (per µL)	267571.7 (84310.9)	256016.9 (77465.0)	-10474.2 (65414.1)	0.0770	-0.4 (25.1)
Total leukocyte count (per µL)	9279.0 (2891.8)	7980.2 (2839.4)	-1290.1 (2868.9)	<0.0001	-10.3 (27.1)
Serum Alkaline phosphatase (IU/L)	93.9 (27.6)	91.1 (30.6)	-2.0 (23.1)	0.3355	0.3 (28.2)
Alanine aminotransferase(U/L)	25.5 (17.5)	24.3 (11.8)	-1.5 (15.6)	0.2998	4.4 (36.9)
Aspartate aminotransferase (U/L)	25.6 (10.4)	24.3 (8.2)	-1.5 (10.4)	0.1200	-1.0 (26.8)
Gamma- glutamyl transferase (U/L)	27.4 (18.3)	24.5 (12.5)	-2.5 (16.5)	0.0921	5.2 (57.8)
Lactate dehydrogenase(U/L)	221.5 (98.0)	216.8 (86.6)	-7.7 (91.6)	0.3523	14.9 (113.2)
Total protein (g/dL)	7.3 (0.6)	7.3 (0.6)	-0.1 (0.6)	0.1349	-0.8 (8.2)
Albumin (g/dL)	4.3 (0.4)	4.2 (0.4)	-0.1 (0.4)	0.0112	-1.8 (9.4)
Total bilirubin (mg/dL)	0.6 (0.3)	0.5 (0.3)	-0.0 (0.2)	0.1665	4.1 (54.4)
Serum creatinine (mg/dL)	0.8 (0.2)	0.8 (0.2)	-0.0 (0.2)	0.6948	1.7 (21.6)

N, total number of patients; n, number of patients; SD, standard deviation.
Note**:** Change from baseline= Post-baseline – Baseline.
% Change from baseline = (Post-baseline – Baseline / Baseline) X 100**.**

Supplementary Table 5: List of ethics committees of participating centers

Sr. No.	Name of principal investigator	Ethics committee name	Ethics committee approval reference number
1	Dr Rohit Kumar	Institutional Ethics Committee at VMMC and Safdarjung Hospital	IEC/VMMC/SJH/Trial/2022-01/CC-226
2	Dr Priti L Meshram	Institutional Ethics Committee at Department of Pulmonary Medicine, Grant Government Medical College and Sir J. J. Group of Hospitals	lEC/Pharm/ CT/ 653 /Dec/2021
3	Dr Venkata Nagarjuna Maturu	Institutional Ethics Committee at Yashoda Hospital	CDT/27/2021
4	Dr Deepak Talwar	Metro Ethical Review Board	64/MERB/2021
5	Dr Murali Mohan	Narayana Health Medical Ethics Committee	S97/2021
6	Dr. Ashish Kumar	Institutional Ethics Committee at Asthma Bhavan	IECAB/2021/175
7	Dr. R Narasimhan	Institutional Ethics Committee - Clinical Studies at Apollo Hospitals	AMH-013/10-2021
8	Dr. Ajay Kumar Verma	Institutional Ethics Committee at King George's Medical University	1841 Ethics/2021
9	Dr. Randeep Guleria	Institute Ethics Committee at All India Institute of Medical Science, New Delhi	IEC-255/04.03.2022, RP-03/2022
10	Dr Ujjwal Parakh	Ethics Committee at Sir Gangaram Hospital	EC/10/21/1967
11	Dr. Janipalli Venkata Praveen	Institutional Ethics Committee at Kinge George Hospital	Not Available
12	Dr. Rahul Sharma	Good Society Ethical Research - Institutional Ethics Committee	GSER/2022/AP/108
13	Dr Rahul Sharma	Institutional Ethics Committee at Fortis Hospital	FHIEC/2022/06
